# Travel Planning Ability in Right Brain-Damaged Patients: Two Case Reports

**DOI:** 10.3389/fnhum.2020.00117

**Published:** 2020-03-31

**Authors:** Alessia Bocchi, Massimiliano Palmiero, Maddalena Boccia, Antonella Di Vita, Cecilia Guariglia, Laura Piccardi

**Affiliations:** ^1^Cognitive and Motor Rehabilitation and Neuroimaging Unit, IRCCS Fondazione Santa Lucia, Rome, Italy; ^2^Psychology Department, Sapienza University of Rome, Rome, Italy; ^3^Department of Human and Social Sciences, University of Bergamo, Bergamo, Italy

**Keywords:** planning, spatial navigation, navigational planning, navigational impairments, travel planning, right brain lesions, topographical orientation

## Abstract

Planning ability is fundamental for goal-directed spatial navigation. Preliminary findings from patients and healthy individuals suggest that travel planning (TP)—namely, navigational planning—can be considered a distinct process from visuospatial planning (VP) ability. To shed light on this distinction, two right brain-damaged patients without hemineglect were compared with a control group on two tasks aimed at testing VP (i.e., Tower of London-16, ToL-16) and TP (i.e., Minefield Task, MFT). The former requires planning the moves to reach the right configuration of three colored beads on three pegs, whereas the latter was opportunely developed to assess TP in the navigational environment when obstacles are present. Specifically, the MFT requires participants to plan a route on a large carpet avoiding some hidden obstacles previously observed. Patient 1 showed lesions encompassing the temporoparietal region and the insula; she performed poorer than the control group on the ToL-16 but showed no deficit on the MFT. Conversely, Patient 2 showed lesions mainly located in the occipitoparietal network of spatial navigation; she performed worse than the control group on the MFT but not on the ToL-16. In both cases performances satisfied the criteria for a classical dissociation, meeting criteria for a double dissociation. These results support the idea that TP is a distinct ability and that it is dissociated from VP skills.

## Background

Planning ability is fundamental for ensuring efficient spatial navigation and it can explain the wide individual differences frequently reported in spatial navigation, both in healthy (Wolbers and Hegarty, 2010; Sharma et al., 2016; Bocchi et al., 2017, 2019) and in clinical populations (Passini et al., 1995; Ciaramelli, 2008). Passini et al. (1995) reported that patients with mild-to-moderate Alzheimer’s Disease failed in reaching a destination in the hospital and this was mainly due to their impaired planning ability. They struggled to build structured plans, binding each sub-goal to the next one (take the elevator to reach the 5^th^ floor) employing a “trial-and-error” strategy. The Prefrontal Cortex (PFC) seems to support the planning ability involved in navigational skills (Ciaramelli, 2008). Indeed, PFC is crucial for several functions of spatial navigation, such as processing of context representations following the navigational goal (Martinet et al., 2011), keeping some spatial information in working memory (Ciaramelli, 2008; Lithfous et al., 2013), planning a path (Spiers, 2008), applying strategies (Dahmani and Bohbot, 2015) and modifying the ongoing plan when detours are required (Boccia et al., 2014a; Spiers and Gilbert, 2015).

Several studies suggested the existence of a specific planning ability devoted to solving navigational tasks (Basso et al., 2006; Cazzato et al., 2010; Martinet et al., 2011; Boccia et al., 2014a; Schacter et al., 2017). This ability has been called in several ways: visuospatial planning (VP; Basso et al., 2006; Cazzato et al., 2010), spatial navigational planning (Martinet et al., 2011; Schacter et al., 2017; Carrieri et al., 2018: Bocchi et al., 2017) or, even, travel planning (TP; Bocchi et al., 2019) that is the term we will adopt thereafter. Martinet et al. (2011) defined TP as the mental evaluation of alternative action-sequences to infer optimal trajectories for reaching a goal, suggesting the dynamic nature of this kind of planning. In this vein, TP could be differentiated from visuospatial planning. Direct evidence of the dissociation between travel and visuospatial planning is not yet available, but some indirect evidence could help in disentangling this issue.

A distinction between the processing of visuospatial and navigational information is detectable in different cognitive domains, being visuospatial information, at least in part, acquired and processed differently from navigational information (Piccardi et al., 2008; Nemmi et al., 2013; Bianchini et al., 2014a). Indeed, the perception of navigational stimuli involved specific cortical areas that are not involved in the perception of other types of visuospatial inputs (Epstein and Kanwisher, 1998). Another distinction refers to memory processing. While visuospatial working memory requires remembering positions in the environment, navigational working memory also requires the continuous updating of our perspective every time a new orientation is provided (e.g., a turn); this would constitute an additional load for the navigational working memory (Nori et al., 2009). Some studies have provided evidence for double dissociations in visuospatial and navigational working memory (Piccardi et al., 2010, 2011). Also, in the mental imagery domain, different kinds of mental images have been described (Guariglia and Pizzamiglio, 2006; Palmiero et al., 2019): topological images are mental representations of stimuli in which the subject can navigate (i.e., rooms, squares, cities, maps, etc.,) and that can be transformed into (or correspond to) cognitive maps of the environment. Non-topological images are mental representations of stimuli, such as a desktop, the interior of a car (Ortigue et al., 2003), single objects or arrays of objects, which can be manipulated but never navigated. Clinical evidence demonstrated the existence of double dissociations between topological and non-topological mental images in brain-damaged patients (Palermo et al., 2010a; Guariglia et al., 2013) as well as differences in mental generation process across the life span (Piccardi et al., 2015); further supports derives from behavioral (Boccia et al., 2014b) and neuroimaging studies (Boccia et al., 2015). Indeed, navigational stimuli are processed differently from common objects (i.e., the clock hands or the map of Italy; Boccia et al., 2014b) in healthy participants; also, these differences are more evident in older individuals (Piccardi et al., 2015). Furthermore, different brain networks support mental imagery of familiar environmental space, geographical space and non-spatial categories, such as faces or clock hands (Boccia et al., 2015, 2019). Neuroimaging evidence also points towards a functional segregation between the processing of navigational information. Indeed, pictures of navigational stimuli (buildings and/or landmarks) activate specific brain regions (i.e., retrosplenial complex, parahippocampal place area, and occipital place area; Aguirre et al., 1998; Epstein and Kanwisher, 1998; Ishai et al., 1999; Dilks et al., 2013) when compared with other categories of objects, such as faces (Ishai et al., 2000; O’Craven and Kanwisher, 2000; Gorno-Tempini and Price, 2001).

Several neural models tried to disentangle the key nodes of TP in the brain. Based on the idea that TP is a complex and multifaceted ability, neural models highlighted the interplay between different brain regions. For example, Martinet et al. (2011) proposed that the interaction between the hippocampus and the PFC yields to the encoding of manifold information pertinent to TP, including prospective coding and distance-to-goal correlates. The hippocampal formation would send the representations of the spatial context to the PFC, which in turn would process such representations according to the current situation. Similarly, Ekstrom et al. (2014) suggested that hippocampal and extra-hippocampal areas (i.e., parahippocampal, retrosplenial, prefrontal and parietal cortices), characterize the neural basis of spatial representations during navigation. According to Spiers and Gilbert (2015), the hippocampal-prefrontal reciprocal interactions would be fundamental when detours require to revise a travel plan.

Finally, the view that travel planning is a distinct ability from visuospatial planning is also supported by the evidence that individuals with developmental topographical disorientation (DTD; Iaria et al., 2009; Bianchini et al., 2010, 2014b; Nemmi et al., 2015; Conson et al., 2018) show impairments in travel planning but not in planning *per se* (Bianchini et al., 2010). Indeed, patients with DTD and good visuospatial planning skills may show a selective deficit in planning a route to reach a destination. In other words, this study suggested that travel planning could be selectively impaired.

Overall, these studies lead to hypothesize that travel and visuospatial planning may be distinct abilities. Despite studies directly testing these differences, especially in brain-damaged patients, are still lacking, it is important to approach such an investigation for both theoretical and clinical implications. It can disclose not only if these processes are dissociated, but also it can be useful for disentangling subtle deficits in travel planning in brain-damaged patients who usually show motor impairments (Mohr and Binder, 2011), and thus may need to set out alternatives ways to blocked-routes. Therefore, in this study we describe patients who performed worse than controls in only one of the two tests tapping TP and VP, showing also greater differences between the two tasks than those expected for the controls, thus meeting the criteria for classical dissociation. The opposite pattern of performance we detected in the two patients provides evidence for a double dissociation between TP and VP skills, for the very first time.

## Case Report

This study was designed following the principles of the Declaration of Helsinki and was approved by the ethical committee of the IRCCS Fondazione Santa Lucia (Protocol number: CE/PROG.670; date: 18th April 2018). Written informed consent was obtained from all participants for participation in the study and the publication of this case report.

Patients underwent an extensive neuropsychological assessment aimed at excluding deficit in general cognitive functioning and visuospatial disorders. After that, they underwent the Mine Field Task (MFT) and the Tower of London (ToL-16) task, to test TP and VP, respectively. For easiness of exposition, neuropsychological assessment and planning evaluation will be divided into subheadings.

### Neuropsychological Assessment and Lesion Description

The neuropsychological assessment included tasks of orientation in time and space (Spinnler and Tognoni, 1987); abstract and/or verbal reasoning (Raven, 1938; Spinnler and Tognoni, 1987); language functions (Ciurli et al., 1996); visuospatial and verbal working memory (Spinnler and Tognoni, 1987), as well as verbal long-term memory, constructive apraxia and attention (Spinnler and Tognoni, 1987; Table 1).

**Table 1:** Neuropsychological Assessment: Spatial orientation, Temporal orientation, Raven’s progressive matrices; Digit span forward, Digit span backward, Rey 15 item memory test immediate recall, delayed recall and recognizing (Rey 1- Rey 2-Rey 3), Story recall test; ca, Constructive apraxia; Corsi Block Tapping Task (Corsi, 1972; Walking Corsi Test, Piccardi et al., 2008; De Nigris et al., 2013), Visual search test.

No.	Spatial orientation	Temporal orientation	Raven	Digits f	Digits b	Rey 1	Rey 2	Rey 3	Story	Ca	CBT	WalCT	Visual Search
1	+++	+++	2	n.a.	n.a.	n.a	n.a.	n.a	3	4	5	6	4
2	+	+	4	4	4	4	4	0	4	4	6	6	3

The Walking Corsi Test (WalCT; Piccardi et al., 2008, 2013; De Nigris et al., 2013) was administered to assess topographic short-term memory in a vista navigational space, namely “the space that can be visually apprehended from a single location or with only little exploratory movements” (Wolbers and Wiener, 2014, p. 3).

Patients also performed a standard battery for evaluating the presence of hemineglect (Pizzamiglio et al., 1989; Table 1). The battery includes: Letter Cancellation Test; Line Cancellation Test; Wundt-Jastrow Area Illusion Test; Sentence reading.

Finally, patients were assessed for perceptual and representational neglect through the Familiar Squares Description Test (Bisiach and Luzzatti, 1978) and the O’Clock Test (Grossi et al., 1989).

**Figure 1:**
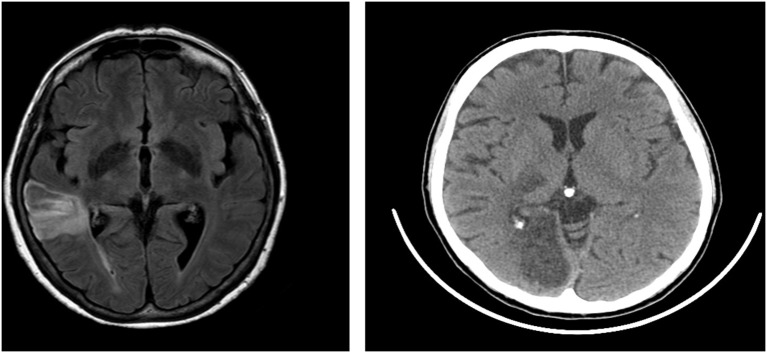
Lesion of Patient 1 (on the left) and Patient 2 (on the right). Patient 1 that showed a lesion in temporoparietal regions with subcortical structures and insula, performed poorly on the visuospatial planning (VP) task (ToL-16) but showed no deficit on the travel planning (TP) task (MFT). Patient 2 that showed a lesion on parieto-occipital areas with the involvement of thalamus performed poorly on the TP task (MFT) but not on the visuospatial task (ToL-16).

Patient 1 was a 65-year-old right-handed woman with 13 years of education, employed as a teacher. Sixty-one days before our examination, she suffered from a stroke involving the right temporal and parietal lobes, extending also to the insula and the subcortical structures (Figure 1). Naming and comprehension abilities were within the normal range. She had neither difficulty in verbal and visuospatial memory tests nor deficits in abstract reasoning. She showed no signs of perceptual or representational neglect.

Patient 2 was a right-handed 51-year-old woman with 13 years of education, employed as a healthcare worker. Fifty-five days before our examination, she suffered from a stroke, involving the right parietal and occipital lobes and the thalamus (Figure 1). Her speech was fluent and informative; her naming and comprehension abilities were intact. The patient did not show difficulty in either verbal or spatial short and long-term memory. She had no deficit in abstract reasoning and did not show signs of perceptual or representational neglect.

Details about lesions (Supplementary Tables S1, S2) and disconnections (Supplementary Table S3) are reported in Supplementary Material, along with the procedure used to derive them. In brief, patient 1 showed a lesion mainly located in the frontal lobe, extending to the parietal and the temporal lobe, as well as disconnection in a wide number of frontal and frontoparietal tracts. Instead, patient 2 showed a lesion of the occipital lobe (including the posterior cingulate/retrosplenial cortex), extending only marginally to the temporal lobe, the basal ganglia, and the cerebellum; she also showed disconnection of these posterior areas and fronto-temporal tracts. Lesion reconstructions are also depicted Supplementary Figure S1.

### Assessment of Planning Abilities

Participants were tested individually in a quiet room. The administration order of the MFT and ToL-16 was counterbalanced across the participants.

#### The Minefield Task (MFT)—TP Task

**Figure 2:**
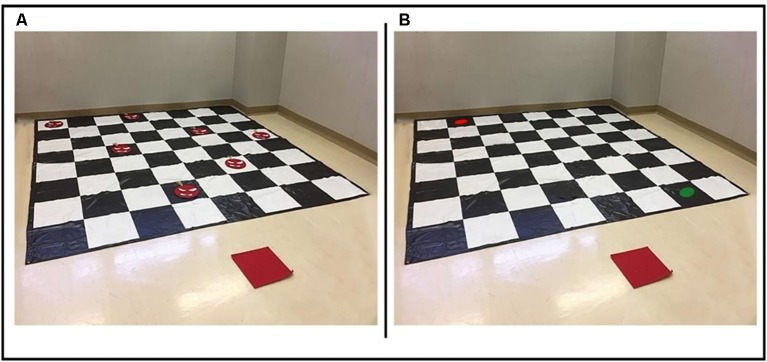
An item from the MFT. **(A)** The chessboard in the acquisition phase; participants were allowed to see the mine locations. **(B)** The chessboard without the mines on it; participants were allowed to see it after being unblindfolded. Green and Red circles indicated the start and the end of the route to plan.

The MFT aims to assess the ability to plan a route on a matrix, avoiding some invisible obstacles (false mines) previously seen for a few seconds. It consisted of a walkable white/black chessboard (8 × 8 matrix, 2.5 × 2.5 m) placed on an empty room (Figure 2). An additional tile was placed out of the matrix (1 meter below the chessboard) to indicate the starting position. Two circles with a 10 cm diameter (one red, one green) were used to indicate the starting and the ending positions of each route. Some “mines” of 15 cm diameter made with red and white felt were placed during the observation phase on the chessboard. The number of mines that could be placed on the matrix ranged from two to nine depending on the trail difficulty. In the first trial, two mines were placed on the chessboard, with the number of mines progressively increasing by one in the successive trials (three mines in the second trial, four in the third and so on). Each trial included two items; therefore, the total number of possible trials was 16.

Participants were placed on the starting position outside the matrix and blindfolded. Two experimenters placed the mines on the matrix. Then, in the observation phase, the eye patch was removed, and participants were required to carefully study the position of the mine on the chessboard. After 10 s, participants were blindfolded again, and the experimenters removed the mines on the chessboard and put the starting and ending point on the chessboard within 10 s. After that, in the testing phase, the blindfold was removed, and the participants had to perform the planned route. Participants moved on the chessboard only vertically and horizontally but not diagonally. Between the starting and ending points, many routes were possible; to avoid that they chose a long, peripherical ride to avoid all the mines, participants were instructed to use the shortest one. To allow testing patients with motor disorders, similarly to the WalCT adopted with patients (De Nigris et al., 2013), participants performed the route from the green circle to the red circle by using a pointer, being careful to avoid the positions in which they had seen the mines in the observation phase. The task began with only two mines on the matrix. If the participant succeeded to avoid them, the second item of the same trial was not presented, and the next trial was administered. On the contrary, if the participant failed, the second item of the same trial was administered. The task was stopped when participants failed to reproduce both items of a given trial. The MFT allowed obtaining a score that corresponded to the number of mines in the longest sequence correctly performed. The maximum score was 9, the minimum was 0.

#### ToL-16 (Shallice, 1982; Boccia et al., 2017a)—VP Task

The ToL-16 was aimed at assessing visuospatial planning ability. Although ToL has long been considered a measure of general planning ability, recent studies have disclosed a visuospatial component in this task (Unterrainer et al., 2004; Franceschi et al., 2007; Cheetham et al., 2012).

The version used in the present study included 16 trials (Boccia et al., 2017a) of increasing difficulty with a maximum number of allowed moves that vary from 2–7. Following Krikorian et al. (1994), the accuracy corresponded to the number of solved problems according to the number of attempts needed to achieve the solution (i.e., 3 = solved at first attempt; 2 = solved at second attempt; 1 = solved at the third attempt; 0 = not solved). Here, the sum of the accuracy at each trial (maximum accuracy = 48) was considered.

### Statistical Analyses and Results

Neuropsychological assessment of the two patients is summarized in Tables s 1, 2. Performances on the experimental tasks of patients and controls are reported in Table 3.

**Table 2:** Patients’ score at the standard battery for evaluating the neglect syndrome (Pizzamiglio et al., 1989).

No.	Left H	Right H	Left-lines	Right-lines	W-J test (unattended responses)	Sentence reading	O’Clock test (LQ)	Square description test (LQ)
1	53/53	51/51	11/11	10/10	0	6/6	−10.34	12.5
2	52/53	51/51	11/11	10/10	0	6/6	0	0

*Left h/Right h: Letter Cancellation Test, left and right (Pizzamiglio et al., 1989). L-Lines: Line Cancellation Test, left and right (Pizzamiglio et al., 1989). W-J Test: Wundt-Jastrow Area Illusion Test (Pizzamiglio et al., 1989). Sentence reading (Pizzamiglio et al., 1989); O’Clock test- Laterality Quotient (Grossi et al., 1989); Familiar Square Description- laterality quotient (Bisiach and Luzzatti, 1978)*.

**Table 3:** Scores of the patients 1 and 2 and the mean scores obtained by the Control Group on the experimental tasks (MFT: Minefield Task and on the ToL-16).

	MFT SCORE	ToL-16 SCORE
CONTROL GROUP	5.1	36.5
PATIENT 1	9	24
PATIENT 2	2	46

We analyzed patients’ performances on the ToL-16 and MFT tasks considering a group of control (Table 1) which included participants with no signs of neurological or psychiatric disorders (10 participants, 3 males; mean age = 57.2 years; SD = 9.1; mean education = 9.1; SD = 3.5). We expected that the difference between the cases’ standardized scores on the two tests was greater than the difference between the same two tests obtained from a control group. The Crawford’s analysis for single cases (Crawford and Howell, 1998; Crawford and Garthwaite, 2002) was applied using the computer program DISSOCSBayes_ES.EXE (Crawford and Garthwaite, 2007), which first tests whether the case’s scores meet the criterion for a deficit on Tasks X and Y (Crawford and Howell, 1998) and then applies the Bayesian Standardized Difference Test (BSDT) to the standardized difference between the case’s scores on Tasks X and Y.

Patient 1 showed a deficit in the ToL-16 task (*t*_(1, 9)_ = −1.8; one-tailed *p* = 0.05) but not in the MFT (*t*_(1, 9)_ = 2.5; one-tailed *p* = 0.01); actually patient 1 scored better than the control group in the MFT. She fulfilled the criteria for a dissociation, putatively a classical dissociation. Patient 2, instead, showed a deficit in the MFT (*t*_(1, 9)_= −2.0; one-tailed *p* = 0.03) but not in the ToL-16 task (*t*_(1, 9)_= 1.4; one-tailed *p* = 0.09). She fulfilled the criteria for a dissociation, putatively a classical dissociation.

The BSDT on the difference between the case’s standardized scores obtained on the ToL-16 task and MFT showed a probability that the standardized difference for a member of the control population would be greater than that of the case showed for patient 1 of 0.00226 (one-tailed), being the effect size (Z-DCC) for the difference between the case and controls (plus 95% Credible Interval): Z-DCC = −3.715 (95% CI = −5.704 to −2.042). The BSDT on the difference between the case’s standardized scores obtained on the ToL-16 task and MFT showed a probability that the standardized difference for a member of the control population would be greater than that of the case shown for patient 2 of 0.00851 (one-tailed), being the Z-DCC for the difference between case and controls (plus 95% Credible Interval): Z-DCC = 2.899 (95% CI = 1.538–4.497).

## Discussion

Dissociations play a key role in building and testing theories in cognitive neuroscience, for instance providing critical support for several models in the field (Dunn and Kirsner, 2003). A classical dissociation (Shallice, 1988) requires that a patient obtained an “impaired” performance on task X, but his/her performance is “not impaired” on task Y (see also Ellis and Young, 1996). Furthermore, Crawford et al. (2003) argued that a further criterion is needed for a classical dissociation: “a comparison of the difference between a patient’s scores on the two tasks of interest to the differences on these tasks observed in the control sample” (Crawford and Garthwaite, 2007, p. 349). This also allows avoiding incorrectly classified cases (Crawford and Garthwaite, 2005, 2006). Following this criterion, this study compared the performances on VP and TP of two right brain-damaged patients without neglect. We provided evidence for a double dissociation between the two types of planning, supporting the idea that they could be considered distinct abilities, involving different cognitive processes that are subtended by, at least partially, different neural bases.

Patient 1, whose performance was good on the MFT, performed poorly on the ToL-16 task, showing impairment in VP and an intact TP. Patient 2 performed poorly on the MFT but not on the ToL-16 task, showing a normal VP and an impairment in TP. These results suggest that only the lesion of Patient 2, which involved the right occipitoparietal lobes, impaired TP. This result allows drawing some conclusions. First of all, Patient 1 showed an adequate level of cognitive functioning. Her lesion and disconnections involved the temporoparietal regions and the insula, but not the PFC; nevertheless, she showed an impairment in the ToL-16 task, in the absence of deficits in TP. This performance can be explained considering that brain regions compromised in Patient 1 contribute to VP (e.g., insular cortices Owen, 1997; Robbins, 1998; van den Heuvel et al., 2003).

Patient 2 showed a globally preserved cognitive profile with performances adequate in all the cognitive domains. She performed worse than the control group on the TP but not on the VP. Interestingly, her lesion encompassed areas of the occipital and the parietal lobe involved in learning positions within navigational vista space (Nemmi et al., 2013) during the WalCT, which is the same space of the MFT. In light of Wolbers and Wiener’s definition (2014; p. 3) of the vista space, Patient 2’s performance could be explained considering the common features of WalCT and MFT and the navigational nature of the MFT. Both the WalCT and MFT require to remember positions in the navigational vista space, to implement a route and to process information available only for a short time. Importantly, during the MFT, differently from the WalCT, this information should be further manipulated and used to perform the task (i.e., avoiding the mines). Furthermore, the MFT requires to decide which route to perform to reach the goal of choosing the shortest one among several alternatives, while the WalCT requires to remember a given route. Thus, a more active involvement of the PFC should be present in the MFT, since planning is less involved in the WalCT. Accordingly, patients performed well within the normal range on the WalCT, supporting the idea that the two tasks tested different aspects of navigation. In other words, it is possible to explain Patient 2’s performance considering that the lesioned areas are important to navigation, for example when positions in the environment should be remembered. These areas could be important either during the WalCT and the MFT considering that both of them take place in the navigational vista space. However, Patient 2’s performance on the WalCT was good, suggesting that these areas could specifically be involved during TP that, unlike the WalCT, requires to further manipulate the spatial information (position in the environment) to plan the right route to the goal. In sum, Patient 1, who showed lesions and disconnections involving more anterior areas (e.g., insular cortex and frontoparietal tracts), was spared on TP but performed worse than controls on VP. Instead, Patient 2, who showed lesions and disconnections involving the occipitoparietal network of navigation, was spared on VP but performed worse than controls on TP, likely due to an impairment in using spatial information to plan the “right” route to the goal.

At date, TP and VP have been considered two aspects of the same planning, sub-served by the same neurocognitive processes. Indeed, both of them may require the correct functioning of PFC (Martinet et al., 2011; Carrieri et al., 2018; Choi et al., 2018) to put together the right sequence of actions to reach a goal; however, TP could be considered a specific planning that shares with VP common processes but also differences. Indeed, Patient 2 who showed a deficit of TP but intact capabilities of VP did not show any lesion of PFC, suggesting that TP involves a network that mostly relies on other brain areas.

The ToL-16 task and the MFT share many processes: working memory useful for maintaining online the final configuration in the ToL-16 task and the position of mines in the MFT; the visual mental imagery necessary to plan the sequence of beads movements in the ToL-16 task and that of steps in the MFT; the planning process itself which put in sequence a series of hand actions in the ToL-16 task and a series of displacements in the MFT; the monitoring process which compares the result of the planning with the desired outcome (namely, the right configuration in the ToL-16 task and the reaching of the goal avoiding mines in the MFT). However, at least two of these processes, i.e., working memory and visual imagery, do not rely on the same brain networks. Indeed, several studies showed that memory in navigational space is subserved by a specific network which, at least partially, differs from that involved in visuospatial memory in no-navigational space (Piccardi et al., 2010; Nemmi et al., 2013). Also, for what attains visual imagery, TP would rely more on topological images (mental representations of environmental stimuli, i.e., rooms, squares, etc., corresponding to cognitive maps of the environment—Guariglia and Pizzamiglio, 2006) rather than on non-topological images. The existence of two different systems processing these two types of mental images has been demonstrated by the observation of double dissociation in right brain-damaged patients (Guariglia et al., 2013) and by neuroimaging studies in healthy participants (Boccia et al., 2015).

Thus, the present double dissociation may be due to the derangement of navigational working memory or to a deficit in the topological imagery deficit which does not affect in any way the VP. This interpretation is consistent with previous lesion locations and disconnections observed in patients with representational neglect restricted to topological mental images (Committeri et al., 2015; Boccia et al., 2018). It is also consistent with findings from DTD patients, who seem to struggle to build a navigational plan even though they still perform well within the normal range on the ToL-16 task (Bianchini et al., 2010).

The double dissociation reported here also suggests that the proper functioning of PFC, although fundamental for planning, it is not sufficient to ensure TP. Indeed, TP likely relies on the cooperation of several areas instead of a specific region, in line with recent neural models including travel planning (Martinet et al., 2011; Ekstrom et al., 2014; Spiers and Gilbert, 2015; Schacter et al., 2017). This is in line with the idea that TP is a complex ability requiring other cognitive processes, such as memory and mental imagery (Byrne et al., 2007; Schacter et al., 2007; Wolbers and Hegarty, 2010; Bocchi et al., 2017, 2019).

To sum up, three key findings emerged from the present case reports. First, lesions in the right occipitoparietal lobes impair the ability to plan a route in the navigational space, even in the absence of lesions in the PFC. Second, TP is likely associated with the parieto-medial temporal lobe network of spatial navigation (Kravitz et al., 2011; Boccia et al., 2014a, 2017b; Sulpizio et al., 2016). Third, and most importantly, a double dissociation exists between VP and TP, suggesting that they involve different brain areas, even sharing some processes.

Despite the importance to describe such a dissociation, the present study has some limitations. For instance, the control group should be increased additional indexes for MFT could be derived, such as planning and execution time. Also, memory for mines position could be investigated in future studies, to disentangle the contribution of memory for positions to a deficit in TP. Finally, even if our patients did not show hemineglect, future studies should investigate the possible association between mental imagery deficits in patients with representational neglect (Palermo et al., 2010b; Guariglia et al., 2013) and TP, to definitively disentangle if the deficit in planning a route is due to an impairment of topological mental images.

Notwithstanding, the present findings are important from both a theoretical and a clinical point of view. On the one hand, they provide the first evidence for a double dissociation between TP and VP skills. On the other hand, knowing that TP can be selectively impaired may be useful for improving rehabilitation programs in brain patients who often show motor impairments (Mohr and Binder, 2011).

## Data Availability Statement

The datasets generated for this study are available on request to the corresponding author.

## Ethics Statement

The study, which involved human participants, was reviewed and approved by Comitato Etico Indipendente della Fondazione Santa Lucia-Santa Lucia Foudation, Via Ardeatina, 306, 00179 Roma. The patients/participants provided their written informed consent to participate in this study.

## Author Contributions

AB, LP, MB, AD, and CG conceived and designed the experiment. AB the collected data. AB and MB analyzed the data and made the lesion mapping. All authors contributed to writing the article.

## Conflict of Interest

The authors declare that the research was conducted in the absence of any commercial or financial relationships that could be construed as a potential conflict of interest.
