# Supplementary material for: Travel Planning Ability in Right Brain-Damaged Patients: Two Case Reports
Source: Front Hum Neurosci. 2020 Mar 31;14:117. doi: 10.3389/fnhum.2020.00117 (PMC7137636; doi:10.3389/fnhum.2020.00117)
Supplement: Supplementary file 1 [file Data_Sheet_1.DOCX]

***Supplementary material***

*Details about lesion location and disconnection*

For each patient, lesion mapping was performed using MRIcron, as it follows: the MNI template was rotated from the standard space to the subject space. Then, the lesion was manually drawn on the reoriented template and subsequently taken back to the MNI space using the inverse rotation (Doricchi 2003). Lesions are graphically reported in Supplementary Figure 1 (Figure S1).


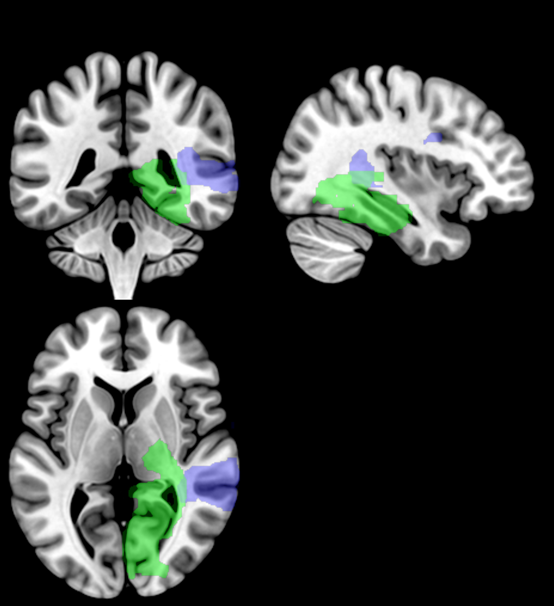


Figure S1 Lesion mapping. Patient 1’s lesion is showed by blue patch, whereas Patient 2’s lesion is showed by green patch

We used Tractotron, as part of the BCB toolkit (<http://www>.brainconnectivitybehaviour.eu/), to identify the tracts that could be affected by the lesion of each patient. Tractotron automatically computes the overlap of each segmented lesion with the map of the tracts. We quantified the probability of the disconnection (Thiebaut De Schotten et al. 2014) and considered disconnected those tracts which showed probability to be disconnected higher than the chance level (probability > 0.5).

Here we report three tables (Table S1, S2 and S3) in order to show descriptive statistics derived from MRIcron about lesion location (Table S1 and S2 for Patient 1 and 2 respectively) and disconnection (Table S3).

**Table S1.** Descriptive statistics derived from MRIcron showing the regions involved in the lesion for Patient 1

| Index | Name | numVox | numVoxNotZero | fracNotZero |
| --- | --- | --- | --- | --- |
| 0 | 0 | 5629168 | 5430 | 0.001 |
| 2 | Precentral_R 2002 | 27058 | 1568 | 0.058 |
| 12 | Frontal_Inf_Oper_R 2302 | 11174 | 1503 | 0.135 |
| 14 | Frontal_Inf_Tri_R 2312 | 17132 | 70 | 0.004 |
| 18 | Rolandic_Oper_R 2332 | 10733 | 54 | 0.005 |
| 38 | Hippocampus_R 4102 | 7606 | 10 | 0.001 |
| 58 | Postcentral_R 6002 | 30652 | 388 | 0.013 |
| 82 | Temporal_Sup_R 8112 | 25258 | 9310 | 0.369 |
| 84 | Temporal_Pole_Sup_R 8122 | 10654 | 3 | 0 |
| 86 | Temporal_Mid_R 8202 | 35484 | 8098 | 0.228 |

**Table S2.** Descriptive statistics derived from MRIcron showing the regions involved in the lesion for Patient 2

| Index | Name | numVox | numVoxNotZero | fracNotZero |
| --- | --- | --- | --- | --- |
| 0 | 0 | 5629168 | 10898 | 0.002 |
| 36 | Cingulum_Post_R 4022 | 2654 | 108 | 0.041 |
| 38 | Hippocampus_R 4102 | 7606 | 2460 | 0.323 |
| 40 | ParaHippocampal_R 4112 | 9028 | 2539 | 0.281 |
| 43 | Calcarine_L 5001 | 18157 | 1332 | 0.073 |
| 44 | Calcarine_R 5002 | 14885 | 11193 | 0.752 |
| 45 | Cuneus_L 5011 | 12133 | 17 | 0.001 |
| 46 | Cuneus_R 5012 | 11323 | 841 | 0.074 |
| 47 | Lingual_L 5021 | 16932 | 420 | 0.025 |
| 48 | Lingual_R 5022 | 18450 | 16130 | 0.874 |
| 50 | Occipital_Sup_R 5102 | 11149 | 238 | 0.021 |
| 52 | Occipital_Mid_R 5202 | 16512 | 313 | 0.019 |
| 54 | Occipital_Inf_R 5302 | 7929 | 1879 | 0.237 |
| 56 | Fusiform_R 5402 | 20227 | 12202 | 0.603 |
| 68 | Precuneus_R 6302 | 26083 | 1372 | 0.053 |
| 72 | Caudate_R 7002 | 7941 | 3 | 0 |
| 74 | Putamen_R 7012 | 8510 | 59 | 0.007 |
| 76 | Pallidum_R 7022 | 2188 | 4 | 0.002 |
| 78 | Thalamus_R 7102 | 8399 | 2561 | 0.305 |
| 90 | Temporal_Inf_R 8302 | 28468 | 753 | 0.026 |
| 92 | Cerebelum_Crus1_R 9002 | 21017 | 19 | 0.001 |
| 96 | Cerebelum_3_R 9022 | 1600 | 129 | 0.081 |
| 98 | Cerebelum_4_5_R 9032 | 6763 | 1997 | 0.295 |
| 100 | Cerebelum_6_R 9042 | 14362 | 1630 | 0.113 |
| 110 | Vermis_3 9110 | 1822 | 38 | 0.021 |
| 111 | Vermis_4_5 9120 | 5324 | 99 | 0.019 |

**Table S3.** Probability of disconnection in patient 1 (P1) and 2 (P2) (only tracts which probability of disconnection were higher than 0 for at least one patient are reported). Tracts significantly disconnected are marked in bold

| N | tract | P1 | P2 | n | tract | P1 | P2 |
| --- | --- | --- | --- | --- | --- | --- | --- |
| 1 | Anterior_Commissure | 0.45 | **0.62** | **33** | Fronto_Insular_tract2_Left | 0 | 0 |
| 2 | Anterior_Thalamic_Projections_Left | 0 | **1** | **34** | Fronto_Insular_tract2_Right | 0.44 | 0 |
| 3 | Anterior_Thalamic_Projections_Right | **0.96** | **1** | **35** | Fronto_Insular_tract3_Left | 0 | 0 |
| 4 | Arcuate_Anterior_Segment_Left | 0 | 0 | **36** | Fronto_Insular_tract3_Right | **0.98** | 0 |
| 5 | Arcuate_Anterior_Segment_Right | **0.78** | 0 | **37** | Fronto_Insular_tract4_Left | 0 | 0 |
| 6 | Arcuate_Long_Segment_Left | 0 | 0 | **38** | Fronto_Insular_tract4_Right | **0.98** | 0 |
| 7 | Arcuate_Long_Segment_Right | **0.58** | 0 | **39** | Fronto_Insular_tract5_Left | 0 | 0 |
| 8 | Arcuate_Posterior_Segment_Left | 0 | 0 | **40** | Fronto_Insular_tract5_Right | **1** | 0 |
| 9 | Arcuate_Posterior_Segment_Right | **0.88** | 0 | **41** | Fronto_Marginal_tract_left | 0 | 0 |
| 10 | Cingulum_Left | 0 | **1** | **42** | Fronto_Marginal_tract_right | 0 | 0 |
| 11 | Cingulum_Left_anterior | 0 | **1** | **43** | Fronto_Striatal_Left | 0 | **0.56** |
| 12 | Cingulum_Left_posterior | 0 | **1** | **44** | Fronto_Striatal_Right | **1** | **1** |
| 13 | Cingulum_Right | **0.88** | **1** | **45** | Handinf_U_tract_Left | 0 | 0 |
| 14 | Cingulum_Right_Anterior | **0.97** | **0.98** | **46** | Handinf_U_tract_Right | 0 | 0 |
| 15 | Cingulum_Right_Posterior | 0 | **1** | **47** | Handmid_U_tract_Left | 0 | 0 |
| 16 | Corpus_callosum | **1** | **1** | **48** | Handmid_U_tract_Right | 0 | 0 |
| 17 | Cortico_Spinal_Left | 0 | **0.62** | **49** | Handsup_U_tract_Left | 0 | 0 |
| 18 | Cortico_Spinal_Right | **1** | **1** | **50** | Handsup_U_tract_Right | 0 | 0 |
| 19 | Face_U_tract_Left | 0 | 0 | **51** | Inferior_Fronto_Occipital_fasciculus_Left | 0 | 0.36 |
| 20 | Face_U_tract_Right | **0.72** | 0 | **52** | Inferior_Fronto_Occipital_fasciculus_Right | **0.98** | **0.76** |
| 21 | Fornix | 0 | **0.85** | **53** | Inferior_Longitudinal_Left | 0 | **0.82** |
| 22 | Frontal_Aslant_Tract_Left | 0 | 0 | **54** | Inferior_Longitudinal_Right | **0.93** | **0.94** |
| 23 | Frontal_Aslant_tract_Right | **1** | 0 | **55** | Optic_Radiations_Left | 0 | **0.54** |
| 24 | Frontal_Commissural | **1** | **0.9** | **56** | Optic_Radiations_Right | **0.61** | **0.93** |
| 25 | Frontal_Inferior_longitudinal_Left | 0 | 0 | **57** | Paracentral_U_tract_Left | 0 | 0 |
| 26 | Frontal_Inferior_longitudinal_Right | **1** | 0 | **58** | Paracentral_U_tract_Right | 0 | 0 |
| 27 | Frontal_Orbito_Polar_Left | 0 | 0 | **59** | Pons_Left | 0 | **0.58** |
| 28 | Frontal_Orbito_Polar_Right | 0 | 0.42 | **60** | Pons_Right | **1** | **1** |
| 29 | Frontal_Superior_Longitudinal_Left | 0 | 0 | **61** | Superior_Londgitudinal_Fasciculus_III_Left | 0 | 0 |
| 30 | Frontal_Superior_Longitudinal_Right | **0.7** | 0 | **62** | Superior_Londgitudinal_Fasciculus_III_Right | **0.99** | 0 |
| 31 | Fronto_Insular_tract1_Left | 0 | 0 | **63** | Superior_Londgitudinal_Fasciculus_II_Left | 0 | 0 |
| 32 | Fronto_Insular_tract1_Right | 0 | 0 | **64** | Superior_Londgitudinal_Fasciculus_II_Right | **0.98** | 0 |
